# The Interdisciplinary Patient Partner Program: Building Better Health Care Professionals through Mentorship with Patients and Families

**DOI:** 10.15694/mep.2018.0000203.1

**Published:** 2018-09-07

**Authors:** Krista Baerg, Krista Trinder, Cathy Cole, Heather Thiessen

**Affiliations:** 1 University of Saskatchewan; 2 Saskatchewan Health Authority - Saskatoon

**Keywords:** attitudes toward patient, client and family-centered care, interprofessional education, experiential learning

## Abstract

This article was migrated. The article was marked as recommended.

**Introduction**: Patient- and Family Centered Care (PFCC) aims to promote collaborative empowering relationships among patients, families, and health care professionals. Best practice for teaching patient- and family-centred care is unknown.

**Methods, Results**: Patient and Family Advisors were matched with an interdisciplinary group of 2-3 students from medicine, pharmacy, and nursing over a five month period to teach PFCC. Advisors provided their journey in the health care system as a basis for further exploration of the 4 pillars of Patient and Family Centered Care. 28 students and 14 Patient and Family Advisors completed the program. Overall, students and advisors were satisfied with the program. Attitudes toward family centeredness were evaluated on a scale of 1 (Strongly Disagree) to 5 (Strongly Agree). Paired samples t-tests were conducted to gauge perceived increases over the program. All items increased significantly with large effect sizes.

**Discussion**: Patient and Family Advisors highlighted the importance of sharing stories and exploring them through dialogue with students as a key factor in the success of the program. The Interdisciplinary Patient Partner Program also reinforced the power of relationship as a learning tool for students. The interdisciplinary nature of this program resulted in additional learning opportunities such as learning about the interdependencies between health care professionals and the importance of an interdisciplinary approach to health care

**Conclusion**: Matching medicine, pharmacy and nursing students with Patient and Family Advisors is an effective way to improve students’ understanding of Patient and Family Centered Care.

## Introduction

Patient- and Family Centered Care (PFCC) aims to promote collaborative empowering relationships among patients, families, and health care professionals. The Institute for Patient- and Family-Centered Care (no date) identifies four pillars of patient- and family-centered care; respect and dignity, information sharing, meaningful participation and collaboration. Practice of patient/client/family/community-centred care is one of 6 domains described within the Canadian Interprofessional Competency Framework (Canadian Interprofessional Health Collaborative, 2010). In the patient and family-centered care environment, patient and family perspectives are listened to and honored, patient and family knowledge, values and beliefs are incorporated into care planning and delivery patient and family participation in care and decision making is supported and patients and families receive timely, complete, accurate, unbiased and useful information. Collaboration is at the organizational level and involves widespread inclusion of patients and families in policy and program development, implementation and evaluation, health care facility design and professional education (Canadian Interprofessional Health Collaborative; Institute for Patient- and Family-Centered Care).

In Canada, organizations such as Health Canada (2001) and the Association of the Faculties of Medicine in Canada (2018) envision a future where medical education is grounded in social accountability and trains physicians to be leaders in addressing the health needs of individuals and their communities. Health care at its heart relies on human interactions with communications and trust between patients and providers. The Crossing the Quality Chasm report by the Institute of Medicine (2001) defined quality of care as being safe, effective, timely, efficient, equitable, and patient -centered. High-quality care is possible only through co-design and partnerships with patients. Patient preferences and patient insights need to be linked to professional knowledge and experience to produce better care ( Baker at al., 2016). Carmen et al. (2013) developed a framework that links a continuum of patient engagement with 3 distinct areas that patients need to be engaged in healthcare (direct care; organizational design and governance; and policy). To achieve this partnership, patients will need to take on much larger roles with clinicians, including the educating physicians on what engagement of patients means. At present, medical students and young physicians in Canada and elsewhere striving to meet this vision are challenged by an absence of opportunities to acquire relevant knowledge and skills ( Jones et al., 2001). Introducing medical students to community practice experience early, as a replacement for or adjunct to existing lecture-based learning, in concert with a greater emphasis on active learning rather than passive observation is the key to begin to teach engagement skills to future physicians ( Bhate and Loh, 2015).

Many patients experience the healthcare system as passive recipients of care, vulnerable, and with a minimal voice in the delivery of their care. In Saskatchewan, the home province within Canada for this research, the Patient First Review (2009) recognizes “With any change to the established and habitual ways of operating, there will be resistance from those more interested in preserving the status quo than responding to the voices of patients and their families. A passion for quality, and willingness to innovate, and the ability to collaborate will be the prerequisites for Saskatchewan health system leaders” (p.55). To change the health system we must also change our approach to education of our physicians to equip them with the tools of engagement with patient. Many events in patients care happen before or after the entry of the healthcare professionals involvement. By giving students the opportunity to hear from and understand patient experiences they see the value in partnering with patients and families and being part of the change for future clinician our healthcare systems may result in a better patient and family experience and more importantly be the catalyst for safer care for the patient.

Best practice for teaching patient- and family-centred care is unknown. Adoption of patient- and family-centered philosophy within a health service organization alone may not ensure learners will acquire the knowledge and skills necessary for effective practice. Current healthcare providers and preceptors, may not have the attitudes, knowledge or skills to teach or model patient- and family-centered care. Organizational change that supports implementation at the care delivery level, such as policy development, staff education and culture change may lag.

## Program Description

A unidisciplinary Family Care Experience program was first implemented over three academic years, from 2009-2010 to 2012-2013. From the beginning the College of Medicine partnered with the Client and Family Centered Care office affiliated with the health authority. The program was initially developed by the physician lead and a medical student from the College of Medicine along with a Patient Advisor and a Client and Family Centered Care Specialist from the health service organization. The program was revised annually based on survey and focus group evaluation undertaken by Educational Support and Development, College of Medicine. In 2014-15 the Interdisciplinary Patient Partner Program was expanded to include pharmacy students and in 2016-17 to include nursing students. Since inception of the unidisciplinary program in 2009, the steering committee and program facilitators have included a physician, a Patient Advisor, and Client and Family Centered Care Specialist. As the program expanded a liaison from each discipline joined the steering committee.

In 2016-17, the program is embedded within and aligns with course level objectives for PFCC Module, Medicine & Society (Med 112/122), Pharmacy 280, and Family Nursing Program. The program is called the Interdisciplinary Patient Partner Program (InterD-3P). Medical students have several options to fulfill experiential learning requirements for the Patient and Family Centered Care Module. The medical college liaison recruits students to participate. The client and family centered care specialist matches students with patient and family advisors. Patient family advisors have extensive health care experience and are recruited by the client and family care specialist from a network of advisers within the health authority. Expectations and time commitments for students and families are clarified prior to enrolment. In accordance with the health service organization’s policy, patient advisors are eligible for an honorarium. Funding support for this educational program is provided by the health service organization (50%) and the other funding was invoiced to each health discipline based on student ratios. After the initial recruitment phase, the program consists of 3 phases: Orientation, Student/Family Meeting Phase and Wrap-Up. Each year the program evaluates students’ attitude change regarding the concepts of PFCC as well as the patient and family advisors impact.

In 2016-17, 28 students and 14 Patient and Family Advisors completed the program. Advisors were matched with an interdisciplinary group of 2-3 students from medicine, pharmacy, and nursing over a five month period. These interdisciplinary groups met their advisor for a facilitated large group orientation and wrap up at the beginning and end of the program. At orientation, a program overview was provided, expectations were clarified and student groups met advisors for the first time. At the wrap-up, students groups were each asked to present key learnings to the whole group then participated in program evaluation. Over term 1 and 2 of the semester, the small groups were instructed to arrange 2-3 meetings with their own advisor for 1-1.5 hours, at a mutually agreed time. A midpoint mixer was coordinated where student groups rotated tables and met 4 new advisors each for 20 minutes to gain additional perspectives (minimum 1 family advisor meeting prior). Students had the option to accompany patient advisors on medical appointments if able and then to discuss the experience.

## Methods

At the end of the program, students (Total N = 28; Pharmacy N = 12, Medicine N = 14, Nursing N = 2) and advisors (N = 11) participated in program evaluation.

### Student Survey

Students completed a survey about their experience in the program. Students were asked to provide reasons for choosing the experience and to indicate whether their expectations were met. Additionally, students answered a question about whether the timelines for the program were reasonable.

Students were also asked to rate the extent to which the experience allowed them to achieve program objectives and their satisfaction with the experience on a scale of 1 (Not at all) to 5 (Very much).

The student survey also contained a Family Centeredness Attitude Scale The scale consisted of 9 items answered on a scale of 1 (Strongly Disagree) to 5 (Strongly Agree). Students were asked to assess themselves both currently and retrospectively to the start of the program. Space was also provided for comments and feedback. The Family Centeredness Attitude Scale was adapted from Institute for Patient- and Family-Centered Care. (2010) *A Checklist for Attitudes about Patients and Families as Advisors.* (accessed June 4, 2011) and utilized the 9 of 14 items related to the health care encounter and the organization.

### Advisor Survey

Advisors were asked to provide reasons for participating in this experience and to indicate whether their expectations were met. They were also asked to suggest supports and/or resources that would be helpful for families in the program.

Advisors were asked to rate their satisfaction with the experience on a scale of 1 (Not at all) to 5 (Very much). They were also asked if the timelines were reasonable. Space was provided for comments and feedback.

### Analysis

Paired samples t-tests were conducted to gauge perceived increases over the program. Effect sizes (Cohen’s d) were calculated as a practical measure of significance where .2 is small, .5 is medium, and .8 is large. Descriptive statistics, such as means, standard deviations, and percentages were also calculated.

## Results

### Student Survey

In response to the question asking reasoning for signing up for the program, students primarily indicated that they wanted to learn more about the patient experience and some students noted the Interprofessional aspects. Representative quotes are included below:

• *Gain patient perspective on healthcare system. Understand barriers to care.*
• *I wanted to see what experiences patients had so that when I go into practice, I don’t make the same mistakes they have experienced.*
• *I saw it as a cool chance to interact with students in other HCP colleges as well as what we bring to the table for patients.*

Twenty-fourstudents (85.7%) said that the program met their expectations and four (14.3%) were not sure. Comments about their expectations being met included the following:

• *As we got to meet with the patient advisor [that] gave us lots of experience about healthcare system, especially when we went with her in the clinic visit.*
• *I learned so much about the journeys some individuals face every single day, and why it is important to be sensitive to all the needs of an individual.*
• *It was above and beyond my expectations. Every time I left a meeting I felt inspired to become a better [health professional] and include a more family centered and interpersonal aspect to my practice.*

All students thought that the timelines were reasonable.

The extent to which students felt that the program allowed them to achieve various objectives are reported in Table 1.

**Table 1.** Student achievement of program objectives

Objective	Mean	SD
Observe the patient/family perspective of the illness.	4.68	.48
Understand how the clients’ feelings are addressed in a patient-centered approach to care.	4.57	.63
Understand the role of the family in the coordination of care.	4.61	.57
Understand the number and type of unanswered questions the client has for their health professionals.	4.21	.69

Overall, students were satisfied with the Family Care experience with a mean satisfaction score of 4.50 (SD = .58).

Students reported that key strengths of the program were having first-hand experiences gaining the perspectives of patients as well as Interprofessional aspects. The aspects that students reported enjoying are reflected in the following comments:

• *“I loved hearing our advisors experience and the perspective of a client. It encouraged me to look at my own clients in a more personal and individual way.”*
• *Loved meeting other [students] from other disciplines. Loved hearing patient stories.*
• *The personal, firsthand experiences. Meeting with the same group/getting to know the group.*

Suggestions for changes to the program included greater Interprofessional representation, guidance on questions for discussion, and additional meetings. This is reflected in the following comments:

• *Guidance questions to lead group discussion in the first meeting or guide us where to go in subsequent meetings.*
• *To get more meeting in the clinical settings with the patients and the physicians.*
• *My group only had 2 students and I feel we lacked voice from the missing student group (nursing).*

### Family Centeredness

Students reported statistically significant increases for all items in the Family Centeredness Attitude scale from the start to end of the program. Means, standard deviations, levels of significance, and effect sizes are reported in Table 2.

**Table 2.** Family Centeredness Attitude Scale: Current and retrospective ratings

	Item	RetrospectiveM	SD	CurrentM	SD	Significance and Effect Size
**Health care encounter**	Patients and family members bring unique perspectives and expertise to the clinical relationship.	3.74	.81	4.85	.36	p < .001, d = 1.77
	I work to create an environment in which patients and families feel supported enough to speak freely.	3.67	.83	4.70	.54	p < .001, d = 1.47
	I listen respectfully to the opinions of patients and family members.	4.22	.75	4.89	.42	p < .001, d = 1.10
	I encourage patients and family members to participate in decision-making at the program and policy level.	3.68	.72	4.75	.44	p < .001, d = 1.79
**Organizational level**	I consistently let colleagues know that I value the insights of patients and families	3.29	.71	4.43	.69	p < .001, d = 1.62
	I believe in the importance of patient and family participation in planning and decision-making at the program and policy level.	3.54	.74	4.79	.42	p < .001, d = 2.08
	I believe that patients and families bring a perspective to a project that no one else can provide.	3.75	1.00	4.86	.36	p < .001, d = 1.48
	I believe that patients and family members can look beyond their own experiences and issues.	3.39	.79	4.36	.83	p < .001, d = 1.20
	I believe that the perspectives and opinions of patients, families, and providers and equally valid in planning and decision-making at the program and policy level.	3.64	.78	4.71	.53	p < .001, d = 1.60

### Advisor Surveys

Participants indicated that their reasons for participating in the program included improving communication within the healthcare system, and reaching students at an early stage of their training. This is reflected in the following comments: *I think getting the students at such an early stage is key to understanding PFCC. I also find it personally rewarding.*

• *I want to improve communication within the health system to increase the chances of recovery for patients especially ones with mental illness.*

Ten respondents agreed that the experience met their expectations and one was not sure. Representative comments include the following:

• *I fully enjoyed meeting the students, getting to know them and their plans - it was wonderful to feel like I was actually making “use” of my sufferings, to benefit others, to be productive.*
• *My students were very receptive to my stories and showed interest and respect.*
• *The Patient Partner Program is invaluable to students and I believe will influence Health Care when they graduate and become part of the system.*

Respondents provided the following comments when asked what support and/or resources would be helpful for families in the program:

• *I think families support families.*
• *I thought the support and package worked very well.*

All respondents agreed that the timelines were reasonable.

Advisors were satisfied with the program this year, with a mean rating of 4.80 (SD = .42).

When asked to describe which aspects of the program they enjoyed, respondents noted spending time with the students and helping them to gain an understanding of the patient experience. This is reflected in the following comments:

• *I love spending time with all my students. Seeing them grow is just so rewarding. I also commit to follow them through.*
• *I was fortunate to have students in all three disciplines. This helped to broaden the experience for all.*
• *“Sharing our story and having the students get really into understanding the lasting impact negative experience can have for patients and their families.”*

Suggestions for improvement included expanding the program to include additional meets and more students from additional professions. This is reflected in the following comments:

• *Expand it to include other first year students from different disciplines e.g., recreation and occupational therapists (there needs to be a program for them in SK).*
• *I like to see us revisit ours students on a yearly basis to see if they’re still understanding PFCC.*
• *More nursing students.*
• *Perhaps new advisors might want to meet with whole advisor group to clarify their interaction with students.*

## Discussion

Patient and Family Advisors highlighted the importance of sharing stories and exploring them through dialogue with students as a key factor in the success of the program. The Interdisciplinary Patient Partner Program also reinforces the power of relationship as a learning tool for students. This program bypasses the need for controlled settings within the health care system to model and teach patient- and family-centeredness. The patient/family advisor is placed in the role of expert advisor and students’ learning is facilitated outside the health care environment. Program participation is early in the medical curriculum before stereotypes and biases are solidified. The program was satisfactory to participants and students reported increased family centeredness. Long-term follow-up with participants would be of value to determine persistence of attitudinal change. As well, study of key differences in learners who choose this program may inform medical schools.

Patient and family centered care is a core competency described within the Canadian National Interprofessional Competency Framework (2010). This program targets discipline-neutral content, rather than discipline specific learning, in an interprofessional context. The interdisciplinary nature of this program resulted in additional learning opportunities such as learning about the interdependencies between health care professionals and the importance of an interdisciplinary approach to health care. This is reflected on student comments pertaining to this as an added benefit to the structure of the program. All comments regarding the interprofessional nature of the program were positive, that this structure enhanced learning and when disciplines were not represented in small groups that opportunities were lost. Additionally, advisors expressed the desire to increase the interprofessional aspect of the program. The explicit focus on patient and family centered care as a common learning goal may have helped the students to see commonalities across disciplines and reduce power differences between disciplines and levels of training.

Female gender, older age, and low socioeconomic status were significant predictors of positive attitudes toward selected items from the Health Beliefs Attitudes Survey associated with patient-centered care ( Hardeman et al., 2015). This program evaluation did not assess program participants’ age, gender or ethnicity, thus further study is required to determine which student populations experience larger shifts in attitude and higher scores. This program was delivered after an introductory lecture on the pillars of PFCC but does not directly assess skills or knowledge to practice PFCC.

Program capacity is dependent on patient family recruitment and student-advisor ratios however program success was tied to the longitudinal nature of the program and the opportunity for relationship development between student, advisors, and within the group. Despite the small group size, difficulty coordinating schedules occurred for some groups and may have been related to the number of disciplines involved.

Advisors expressed many different motivations for participation, mostly to impact future care within the organization and to find meaning in some of the medical adverse experiences they had within the system. Alignment with the health service organization may be key to patient/family willingness to participate and provided an “arm’s length” opportunity for recruitment of experienced patient advisors orientated to principles of PFCC rather than recruitment by the clinician teachers. Further exploration of factors in patient family willingness to participate would inform other programs dependent on advisors. Lastly, advisors valued access to other advisors for mentorship which occurred at orientation and informally during large group meetings. The Client and Family Care Specialist followed up with the advisors at midpoint and was available throughout the program for consultation.

## Conclusion

The program had positive learning outcomes for the students and met expectations for students, patients and families. This program evaluation demonstrates that when medicine, pharmacy, and nursing students meet directly with Patient Advisors to discuss the patient experience within a PFCC framework, they experience a positive shift in attitude towards Patient and Family Centered Care. As a result of the success of this program the organizers aim to expand this program to include more students as well as more health care disciplines. Continued involvement of a patient and family care specialist is vital for successful recruitment of and follow-through with patient and family advisors. Features of the program that impact satisfaction for both students and advisors include clear student and family expectations, timely follow-up with students and families to identify concerns and attention to feelings related to forming and closing the student family relationships.

## Take Home Messages

Matching medicine, pharmacy and nursing students with Patient and Family Advisors and specific learning objectives is an effective way to improve students’ attitudes toward Patient and Family Centered Care.

## Notes On Contributors

KB, CC, HT made substantial contributions to the design of the work. KT contributed to collection of the data performed the statistical analyses. All authors contributed to data interpretation. KB and KT drafted the manuscript. All authors critically revised the manuscript and contributed to the scientific discussion of the data.
