# Supplementary material for: The Interdisciplinary Patient Partner Program: Building Better Health Care Professionals through Mentorship with Patients and Families
Source: MedEdPublish (2016). 2018 Sep 7;7:203. [Version 1] doi: 10.15694/mep.2018.0000203.1 (PMC10701826; doi:10.15694/mep.2018.0000203.1)
Supplement: Peer review history

# Reviewer report: Reviewer response for version 1

**DOI:** 10.21956/mep.19672.r29378

**Published:** 2019-03-15

**Authors:** Richard Hays

**Affiliations:** 1 James Cook University

This review has been migrated. The reviewer awarded 4 stars out of 5 This is a well written and detailed description of an interesting medical education strategy in Canada. It is similar to a successful strategy at one Australian University, although that is medical students only rather than Interprofessional. The evaluation is positive, but more on the practicalities would be helpful for others considering similar strategies. For example, how does this fit into the students’ schedule, which can be very busy? How do clinical faculty view the strategy and will they support attendance if it distracts from seeing ‘real’ patients? The paper should be of interest to all clinical medical educators, as this is a way of broadening the clinical exposure of medical students as well as providing the patients’ perspectives on health care.

Reviewer Expertise:

NA

No decision status is available

# Reviewer report: Reviewer response for version 1

**DOI:** 10.21956/mep.19672.r29377

**Published:** 2018-09-11

**Authors:** Tripti Srivastava

**Affiliations:** 1 Datta Meghe Institute of Medical Sciences Deemed University

This review has been migrated. The reviewer awarded 3 stars out of 5 Congratulation to the authors for this work! Besides being the most appropriate approach, Patient - Centered care, is the only answer to the rising distrust of society towards health care profession. The manuscript embodies an interdisciplinary health care team with a novel concept of Patient/family advisers acting as links between patient and learners in Health professions. Though the authors have categorically included only the learners perception ( as a part of program evaluation) about the PFCC framework within this manuscript ; it would be interesting to know its actual impact in terms of learner competencies, patient compliance and outcome.

Reviewer Expertise:

NA

No decision status is available
